# Study of TiO_2_/Ti4O_7_ photo-anodes inserted in an activated carbon packed bed cathode: Towards the development of 3D-type photo-electro-Fenton reactors for water treatment

**DOI:** 10.1016/j.electacta.2020.135972

**Published:** 2020-04-20

**Authors:** V. Becerril-Estrada, I. Robles, C. Martínez-Sánchez, Luis A. Godínez

**Affiliations:** a Centro de Investigación y Desarrollo Tecnológico en Electroquímica S. C., Parque Tecnológico Querétaro, 76703, Sanfandila, Pedro Escobedo, Querétaro, Mexico; b CONACYT – Centro de Investigación y Desarrollo Tecnológico en Electroquímica, Querétaro, Mexico

**Keywords:** TiO_2_/Ti_4_O_7_, Magneli, Photoanode, Advanced oxidation processes, Electro-Fenton

## Abstract

In this work, commercially available Polymethyl-meta-acrylate (PMMA) spectroscopy cells were modified on the external walls with films of TiO_2_, Ti_4_O_7_ or TiO_2_/Ti_4_O_7_ mixtures. Film characterization was carried out using SEM and UV–vis spectroscopy. The results of photocatalytic (PC), electro-oxidation (EO), and photoelectrochemical (PEC) experiments on the decolorization of a methyl orange (MO) model dye solution showed that while anatase provides better photocatalytic properties and the partially reduced Ti_4_O_7_ larger electronic conductivity, the TiO_2_/Ti_4_O_7_ composite film behaves as a semiconductor substrate that combines the advantages of both materials (for PEC experiments for instance, decolorization values for the model dye solution using TiO_2_, Ti_4_O_7_ and a TiO_2_/Ti_4_O_7_ mixed film, corresponded to 35%, 46% and 53%, respectively). In order to test this film as an effective photoanode material in a 3-D type reactor for water treatment processes, a TiO_2_/Ti_4_O_7_ modified PMMA spectroscopy cell was inserted in an activated carbon (AC) bed so that the semiconductor material could be illuminated using an external UV source positioned inside the PMMA cell. The connected AC particles that were previously saturated with MO dye were used as cathode sites for the oxygen reduction reaction so that the photoelectrochemical reactions that take place in the anode could be complemented with coupled electro-Fenton processes in the cathode. As expected, the combination resulted in an effective decolorization of the dye solution that results from a complex combination of processes. The experimental decolorization data was successfully fitted to a pseudo-first order kinetic model so that a deeper understanding of the contribution of each process in the reactor could be obtained.

## Graphical abstract

**Figure:**
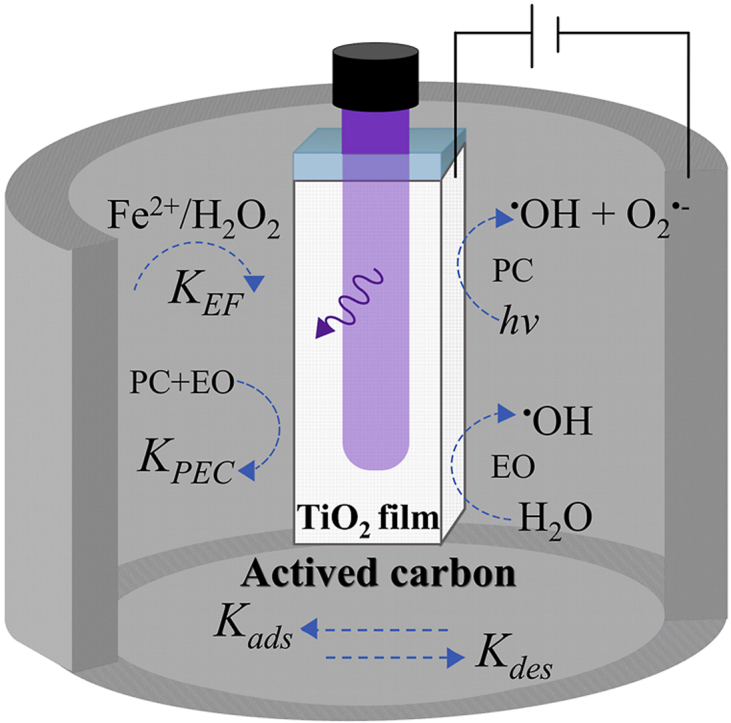

## 1 Introduction

Advanced oxidation processes (AOP) are with no doubt some of the most promising approaches for the treatment of wastewater containing recalcitrant pollutants due to their speed and effectiveness. These processes are characterized by the generation and use of the ^•^OH radical species which is a short-lived, non-selecting and powerful oxidant species that reacts with almost any organic contaminant. There are several AOPs; i.e., numerous ways in which the ^•^OH radical can be produced and used [1,2]. Among these, electrochemical- and photo-assisted AOPs are particularly interesting since electric and radiation stimuli can be simultaneously applied to the system and combined on the surface of an electrode in a synergistic fashion [3]. In this way, electro-oxidation (EO), photocatalytic (PC), photoelectrochemical (PEC), electro-Fenton (EF) and photo-electro-Fenton (PEF) processes are all ^•^OH radical generation techniques [[4], [5], [6], [7]] in which electricity, light or an electrochemically or photo-electrochemically produced mixture of H_2_O_2_ and Fe(II) species, are employed to produce a highly oxidant environment on the surface of either the anode, the cathode, or both.

In photocatalytic as well as in photoelectrochemical studies, the most popular anode material is titanium oxide (TiO_2_) [[8], [9], [10]]. This compound has several advantages such as high photocatalytic activity [11,12], reasonable price, chemical stability and low toxicity [13]. Commercially available TiO_2_, is usually composed of a mixture of anatase and rutile (80-20%) [14] and it is well known that the former is characterized by a higher photocatalytic activity than the later [15].

On the other hand, Magneli phases are a sub-stoichiometric titanium oxides that are described by the generic formula Ti_n_O_2n-1_, where *n* is an integer number between 4 and 10.

Among the different partially reduced degrees that can be found for magneli, Ti_4_O_7_ is not only common and commercially available but also characterized by a high electric conductivity (about 1500 S cm^−1^) that is similar to that of graphite [16]. There are some other interesting properties of Ti_4_O_7_ such as its high chemical stability, hardness and low toxicity [17] that have resulted in its use in batteries, fuel cell stands and photo-catalytic substrates [18]. In addition, Ti_4_O_7_ has been reported to show a high potential for oxygen evolution (2.5 V v*s* RHE) [16] and therefore, the potential use of magneli as an anode material for EO processes, has called the attention of several research groups around the world [19].

Depending on the nature of the excitation signal (light, electric polarization, or both), it is often convenient to immobilize TiO_2_ or Ti_4_O_7_ in either non-conducting [20,21] or conducting [[22], [23], [24], [25], [26], [27], [28], [29]] substrates. In the specific case of photo-anodes, optic fibers have been chemically treated and surface modified with TiO_2_ so that the electrode can be illuminated from within the optic fiber [30,31]. In spite of the promising results of these studies, the SiO_2_ optic fibers that have been employed are fragile and therefore, a good alternative is using plastic cuvettes of a transparent, low cost and easy handling material such as polymethyl-meta-acrylate (PMMA) [[32], [33], [34]]. The advantage of preparing this type of photoanodes is that in principle, the modified cuvettes illuminated from the interior can be inserted in 3D cathodes [35,36]. A good example of this type of electrodes are activated carbon (AC) packed beds which constitute a network of electrically interconnected adsorbent particles on which electrochemical reactions take place [37]. The use of 3D electrodes substantially increases the surface/volume ratio in electrochemical cells, thus improving the efficiency of 3D reactors as compared to that of traditional 2D reactors [38].

In this context, we are hereby presenting the results of a study divided in two parts. In the first one, commercially available PMMA spectroscopy cells were modified with films of either TiO_2_, Ti_4_O_7_, or a mixture of both, and the photo- and photo-electrocatalytic activity of the resulting electrodes was measured and compared. In the second part, the best performing films were introduced in a polarized AC packed bed so that a 3D photoelectrochemical reactor could be tested and characterized as an alternative and promising approach for the development of efficient and cost-competitive electrochemical AOP based reactors for water treatment.

## 2 Experimental section

### 2.1 Chemicals

All chemicals employed in this work, were of analytical grade. Methyl orange (MO), KNO_3_, FeSO_4_·7H_2_O and acetone were purchased from Hycel, Fermont, JT Baker and Sigma, respectively. While, TiO_2_ was obtained from Sigma Aldrich, Ti_4_O_7_ was purchased from Magneli Materials-LLC. All solutions were prepared with ultra-pure deionized water (18 MΩ cm) and when required, a HNO_3_ concentrated solution obtained from Merck was employed to acidify the solution. pH measurements were carried out using a Thermo Scientific Orion Star, A211, apparatus. Lignitic Activated carbon (AC) was obtained from Clarimex (mesh size between 1.0 and 1.4 mm) and used as received.

### 2.2 PMMA cell modification with films of TiO_2_, Ti_4_O_7_ and TiO_2_/Ti_4_O_7_

Commercial 4.5 mL PMMA cuvettes used for UV–vis spectroscopy analysis having flat polymeric 1 × 4.5 cm^2^ sides and a 1 × 1 cm^2^ bottom, were modified with the semiconductor material using a silicon-based commercial glue that was previously diluted with acetone (3.5 g of glue + 15 mL of acetone). In this way, 0.39 g of either TiO_2_, Ti_4_O_7_ or a 1:1 mixture were evenly spread and fixed with the diluted glue on the 4 sides of the PMMA cuvette. The modified cells were dried in an oven at 303 K for 24 h and while the mass of semiconductor material was in all cases 0.39 g, the corresponding thickness was 200 μm.

### 2.3 Semiconductor film characterization

Films anchored on the PMMA modified cells, were characterized using Scanning Electron Microscopy (SEM) and UV–vis Spectroscopy. While SEM measurements were carried out using a (JOEL JSM-5400 L) microscope equipped with an X ray energy dispersion accessory, the UV–vis spectroscopy experiments were performed with an UV-Vis-NIR Agilent Cary 5000, apparatus. Using this spectrophotometer, band gap semiconductor assessment was made using the Kubelka-Munk approach [39].

### 2.4 Electrochemical and photocatalytic dye degradation experiments with semiconductor modified PMMA substrates

**Fig. 1:**
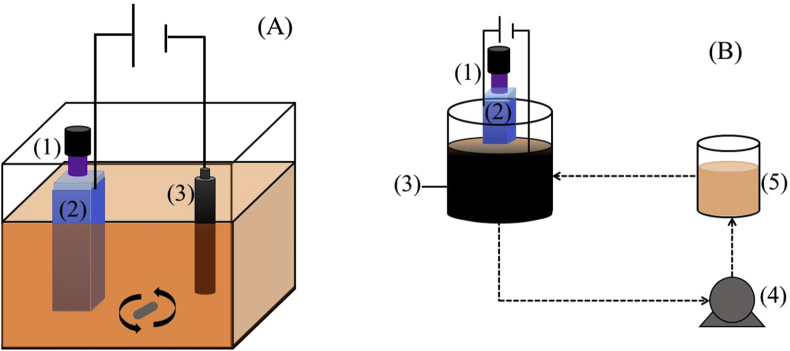
(A) Electrochemical cell arrangement for PC, EO and PEC experiments and (B) 3D reactor for PEC′ and EF/PEC′ MO decolorization tests. (1) UV lamp, λ = 365 nm, (2) Anode consisting on a PMMA cuvette modified with the semiconductor films under study, (3) cathode consisting on -A, graphite rod and -B, AC packed bed, (4) peristaltic pump and (5) dye solution containing tank.

As can be seen in Fig. 1-A, the experimental cell to carry out PC, EO and PEC experiments, consisted on a 100 mL glass flask partially filled with a MO dye containing solution in which the semiconductor modified PMMA cuvette was immersed. For polarization experiments, the semiconductor film supported on the polymeric substrate was connected to a power source (Novak Technologies DCE 10/24–9) which in turn, was coupled to a graphite rod GSP-250 (ø = 1.2 cm). The electrolytic medium consisted on 50 mL of a MO dye solution, 2.5 × 10^−5^ M and KNO_3_ 0.1 M, which was maintained under constant magnetic stirring at pH 3.0. In these experiments, potential differences of 1, 2 and 3 V between these two electrodes were applied for 60 min and samples for dye analysis were extracted from the system every 10 min.

For PC experiments, a pencil-type UV Hg lamp (Spectroline 36–380, λ = 365 nm) was positioned inside the modified PMMA cuvette (see Fig. 1-A). Electromagnetic irradiation was continuously applied for 60 min and every 10 min a sample was taken out from the solution in order to determine the concentration of the dye by means of a standard absorbance measurement.

For PEC experiments, the setup consisted on a system in which polarization and UV radiation were simultaneously applied to the semiconductor modified PMMA electrode.

### 2.5 Dye decolorization performance on a 3-D type AOP-PEC reactor

Fig. 1-B shows the arrangement of a photo-assisted electro-Fenton reactor that works in re-circulation mode (110 mL min^−1^) and in which the semiconductor modified PMMA cuvette (anode) is inserted in an AC packed bed (0.5 g cm^−3^, cathode) previously saturated with the MO dye. Saturation conditions were achieved by exposing the AC sample to a MO saturated aqueous solution for 72 h under stirring. The corresponding saturation adsorption capacity of AC with MO dye (29.3 mg g^−1^) was obtained from the difference of absorbance measurements of the dye solution before and after saturation took place. Using the arrangement in Fig. 1-B, photoelectrochemical (PEC′ to differentiate from PEC experiments in the cell shown in Fig. 1-A), and EF/PEC’ experiments were performed applying a potential difference of 3 V and introducing a pencil-type UV lamp (λ = 365 nm) inside the PMMA modified cuvette.

In these experiments, the electrolyte to be treated consisted on 75 mL of a MO solution (5 × 10^−5^ M) also containing KNO_3_ 0.1 M at pH 3. For EF/PEC’ experiments, the dye contaminated solution contained 5.5 mg L^−1^ of FeSO_4_ and was maintained under oxygen saturation conditions so that the Fenton mixture could be produced upon H_2_O_2_ electro-generation at the cathode surface.

As previously mentioned, the change in MO absorbance (λ = 505 nm) was measured in sample solutions taken out from the reactor at 10 min time intervals using a Genesys 10 S Thermo Scientific spectrophotometer.

## 3 Results

### 3.1 Semiconductor film characterization

**Fig. 2:**
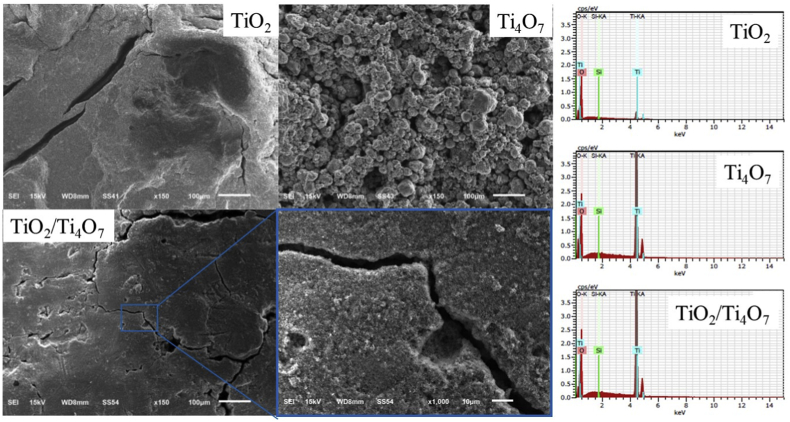
SEM images and EDS spectra of TiO_2_, Ti_4_O_7_ and TiO_2_/Ti_4_O_7_ films prepared on the surface of PMMA substrates.

Fig. 2 shows the morphology of the TiO_2_, Ti_4_O_7_ and the 1:1 TiO_2_/Ti_4_O_7_ films under study. For the TiO_2_ film, a relatively dense structure that loses its consistency at the outermost layers, can be observed. As has been previously discussed in the literature [40], the cracks of ∼20–25 μm in the surface structure have been suggested to be related to a decrease in the TiO_2_ interparticle forces as the film becomes thick. This morphology is quite different to that obtained for Ti_4_O_7_ (see Fig. 2). Inspection of the relevant image shows a disperse and porous structure with particle aggregates of about 50 μm; a feature that should be reflected by a more homogenous film, characterized by a large roughness factor.

The TiO_2_/Ti_4_O_7_ film on the other hand, shows mixed morphology features. In this way, the image shown in Fig. 2 reveals not only a surface structure characterized by a homogenous film with smaller surface cracks (about 10 μm), but also the absence of distinguishable particle aggregates. This is probably due to the better interparticle interaction shown by Ti_4_O_7_ when compared to that displayed by TiO_2_.

Fig. 2 also shows the EDS spectra of the three films under study. As expected, each spectrum shows signals for titanium, silicon and oxygen. It is also interesting to note that in addition to the peaks positioned at energies <3 KeV, the presence of Ti (<IV) in Ti_4_O_7_ and in TiO_2_/Ti_4_O_7_ can be readily identified by the relative intensity observed in the signal at 4.5 KeV.

**Fig. 3:**
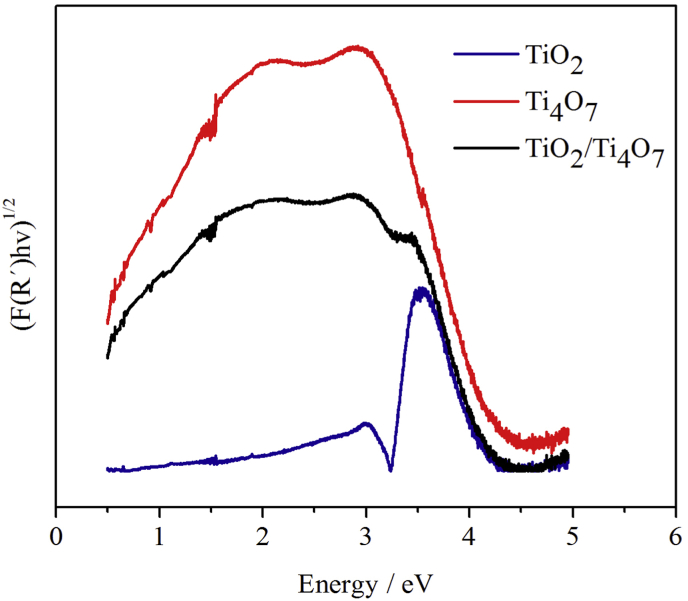
Kubelka-Munk representation of the diffuse reflectance spectroscopy responses for TiO_2_, Ti_4_O_7_ and the TiO_2_/Ti_4_O_7_ films.

Film characterization was also carried out in terms of the light absorption properties of the surface confined materials. As can be seen in Fig. 3, the data corresponding to diffuse reflectance spectroscopic measurements that are plotted following the Kubelka-Munk model [39], shows that the band gap (*Eg*) for TiO_2_ is 3.24 eV; a value that is close to that reported in the literature (3.2 eV) [41]. In this context, a maximum absorption band could not be identified for Ti_4_O_7_. This observation is consistent with reports that point out that although magneli is a reasonable electronic conductor, its performance is quite limited as a photocatalytic material when compared to TiO_2_ [42]. The TiO_2_/Ti_4_O_7_ mixed film on the other hand shows intermediate properties, i.e., the response signal is located between the spectra of TiO_2_ and of Ti_4_O_7_.

### 3.2 Photo-catalytic activity of the semiconductor films

**Fig. 4:**
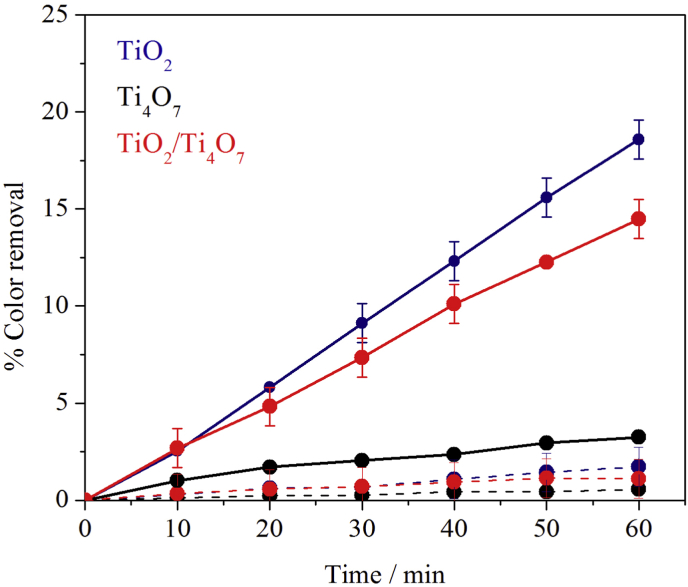
Decolorization kinetics of a 2.5 × 10^−5^ M solution of MO in the absence (- - -) and in the presence (^__^) of UV light (λ = 365 nm) at pH 3, using TiO_2_, Ti_4_O_7_ and TiO_2_/Ti_4_O_7_ semiconductor films.

The photocatalytic activity of the three films under study was tested as described in the experimental section. In this way, the kinetics of dye decolorization was measured following the absorbance decrease (λ = 505 nm) of a MO solution (initial concentration of 2.5 × 10^−5^ M) at pH 3. Control experiments in the absence of UV radiation were first performed in order to assess the adsorption capacity of the electrodes and as can be seen in the dotted lines of Fig. 4, the observed decolorization was in all cases negligible (<2%). Introducing UV radiation results in the continuous line responses shown in Fig. 4. Inspection of the corresponding data shows that while the TiO_2_ and Ti_4_O_7_ films give the best and worst photocatalytic degradation performances (19 and 3% after 60 min), the mixed TiO_2_/Ti_4_O_7_ semiconductor material shows an intermediate behavior (14%), which is in fact closer to that of TiO_2_.

These results support previous observations that point out that the photocatalytic degradation of dye molecules using semiconductor materials, depends not only on the photo-induced generation of hole/electron pairs (h^+^)/(e^−^) but also on the competition of these species to react with the pollutant molecules and with nearby oppositely charged carriers. In addition, different oxidant species can be formed as well. According to equations (1), (2), (3)) [43,44] the photogenerated electrons and holes (see equation (1)) can react with either oxygen or water molecules to produce O_2_^•-^ radical anions in the former and surface adsorbed ^•^OH radicals in the latter case (see equations (2), (3)), respectively).

(1) TiO_2_ + hv → e^−^_CB_ + h^+^_VB_

(2) e^−^_CB_ + O_2_ → O_2_^•-^

(3) h^+^_VB_ + H_2_O → ^•^OH + H^+^

### 3.3 Electro- and photo-electrocatalytic tests

Consistent with the previous set of experiments, the TiO_2_, Ti_4_O_7_ and TiO_2_/Ti_4_O_7_ mixed films were tested by following the kinetics of dye decolorization of a MO solution under polarization conditions as described in the experimental section. In all cases, the potential was applied in such a way that the semiconductor film was the anode and a graphite rod worked as the cathode. Since surface adsorbed ^•^OH radicals can be electrochemically produced (see equation (4)) [45], the experiments were performed applying 1, 2 and 3 V between the two electrodes.

(4) M + H_2_O → MO_x_(^•^OH) + H^+^ + e^−^

**Fig. 5:**
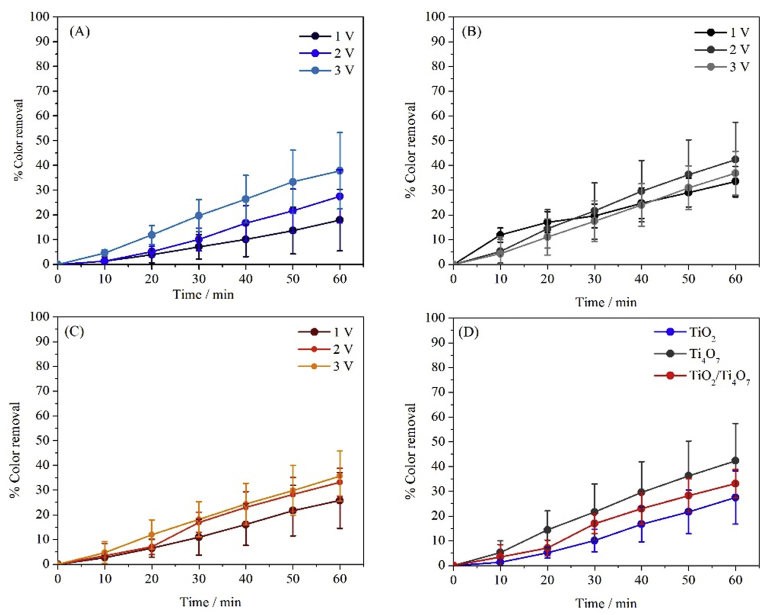
EO induced decolorization curves of 2.5 × 10^−5^ M, MO solutions, using (A) TiO_2_, (B) Ti_4_O_7_ and (C) TiO_2_/Ti_4_O_7_, PMMA modified cuvettes and applying different potentials. (D) Shows a comparison of the MO decolorization kinetics induced by 2 V polarization for each one of the three films under study.

In Fig. 5 the results of the evolution of the decolorization of the dye-contaminated solution using the different semiconductor materials and the different polarization potential values are presented. From the data in this Figure two important observations stand out. On one hand, it is possible to see that within the experimental window the applied voltage is proportional to the rate of decolorization (see Fig. 5-A to -C) and therefore, to the maximum amount of color disappearance that can be measured at a given time (see for example decolorization percentages after 60 min for TiO_2_ in Fig. 5-A, 19 %, 24% and 39% for 1, 2 and 3 V, respectively). Relative to the second observation, comparison of the extent of decolorization using the three different films under study suggests that as opposed to what was observed for PC experiments, the best performance is given by Ti_4_O_7_, the worst is offered by TiO_2_ and the mixture TiO_2_/Ti_4_O_7_ is characterized by an experimental response that can be positioned between those associated to the other two materials (see Fig. 5-D).

The next step, consisted in testing the performance of the three semiconductor films under simultaneous electric and light stimulation. In this way, PEC experiments were carried out as described in the experimental section and as expected, the obtained responses reflected a combination of those previously observed for EO and PC experiments.

**Fig. 6:**
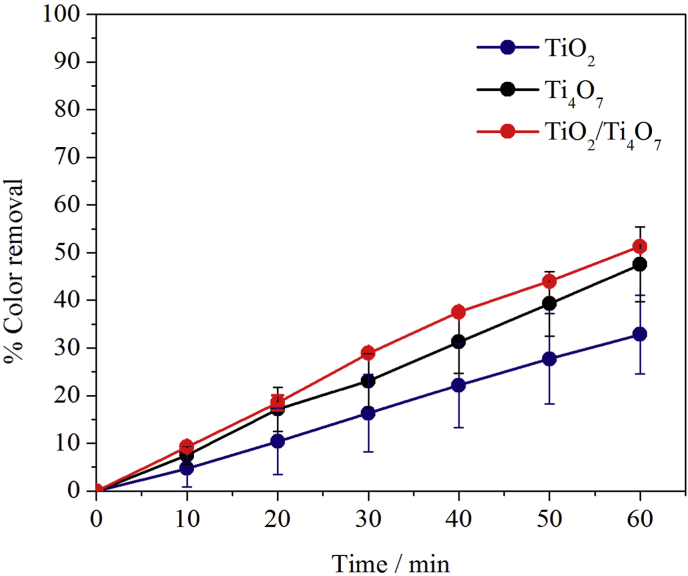
PEC induced decolorization curves of MO solutions using TiO_2_, Ti_4_O_7_ and TiO_2_/Ti_4_O_7_ semiconductor films. Initial MO concentration 2.5 × 10^−5^ M, applied potential 3 V and UV light with λ = 365 nm.

As can be seen from the relevant data shown in Fig. 6, two main differences can be readily identified. First, the decolorization percentages after 60 min are larger for PEC than for the other two approaches (for example, 53% for PEC using TiO_2_/Ti_4_O_7_ *vs* 43 and 19% for EO and PC using Ti_4_O_7_ and TiO_2_, respectively), and comparable to the data of PEC experiments published by other authors [[46], [47], [48]]. The second difference is that, as opposed to what was observed for PC and EO, the best performing semiconductor material is the TiO_2_/Ti_4_O_7_ composite film. As can be seen from the experimental results in Fig. 6 and from equations (1), (2), (3), (4)), the combination of UV irradiation and electric polarization favors the performance of the semiconductor film that not only can efficiently absorb photons and generate (h^+^)/(e^−^) pairs, but that can also be effectively polarized so that charge carrier recombination is hindered and dye-consuming degradation reactions can be promoted [49,50]. In other words, the effective photo-anodic performance of the TiO_2_/Ti_4_O_7_ film observed in Fig. 6 can be explained by the combination of the characteristic photocatalytic and electrochemical activities that were observed for TiO_2_ in the PC and for Ti_4_O_7_ in EO experiments.

### 3.4 Testing a TiO_2_/Ti_4_O_7_ mixed film in a photo-electro-Fenton 3D-type reactor

Considering that the mixed TiO_2_/Ti_4_O_7_ composite was the most effective film using a PEC approach, the 3D-type reactor system shown in Fig. 1-B was set up using the TiO_2_/Ti_4_O_7_-modified PMMA cuvette as anode, an AC packed bed (0.5 g cm^−2^) as cathode and a UV lamp and a power source to provide electromagnetic radiation and electric potential stimuli, respectively. It is important to point out that in order to avoid decolorization effects due to AC adsorption, the cathode material was previously saturated with MO by exposing the material to a dye saturated solution for 72 h. Therefore, in the experiments corresponding to the system in Fig. 1-B, the initial concentration of the MO dye was 5 × 10^−5^ M, the voltage applied by the power source was 3 V and the UV lamp inserted in the PMMA cuvette provided continuous radiation at λ = 365 nm and the reactor used previously saturated AC.

It is also important to note that since the integral processes to be explored considered not only the anodic dye-degradation reactions but also the cathodic effect in the 3D reactor, the addition of 5.5 mg L^−1^ of FeSO_4_ to the electrolytic solution was carried out. As can be seen by the reactions described in equation (5), the electrochemical generation of H_2_O_2_ (by means of oxygen reduction at the polarized AC particle surface) in the presence of Fe(II) cations gives rise to the well-known Fenton mixture which in turn, readily reacts as described by equation (6) to produce the powerful ^•^OH radical [51]. It is also important to note that as equation (7) points out, the cathodic environment at the carbon-solution interphase during the production of ^•^OH radicals is also responsible for the regeneration of Fe(II) from Fe(III); thus effectively sustaining the oxidant electro-generation conditions in the so-called electro-Fenton process (EF) [52].

(5) $$O_{2}+2H^{+}+2e^{-}\to H_{2}O_{2}$$

(6) $$Fe^{2+}+H_{2}O_{2}\to Fe^{3+}{+}^{•}OH+OH^{-}$$

(7) $$Fe^{3+}+e^{-}\to Fe^{2+}$$

Although photo-assisted electro-Fenton processes exists and are an important class of AOPs [53], it is important to emphasize that incorporation of UV light in the experiments using this reactor, will only affect the performance of the anode. Therefore, in order to avoid confusion with the experiments described in the previous section (see for instance the discussion of PEC in Fig. 6), the PEC process in this part of the work will be labeled as PEC’. In this way, while dye decolorization in the polarized and illuminated semiconductor film immersed in the AC packed bed cathode will be marked as PEC′, the EF/PEC′ process will stand for the combination of the photoelectrochemical contribution in the anode (PEC’) and an electro-Fenton process (EF) taking place in the vicinity of the cathode surface (see equations (5), (6))).

**Fig. 7:**
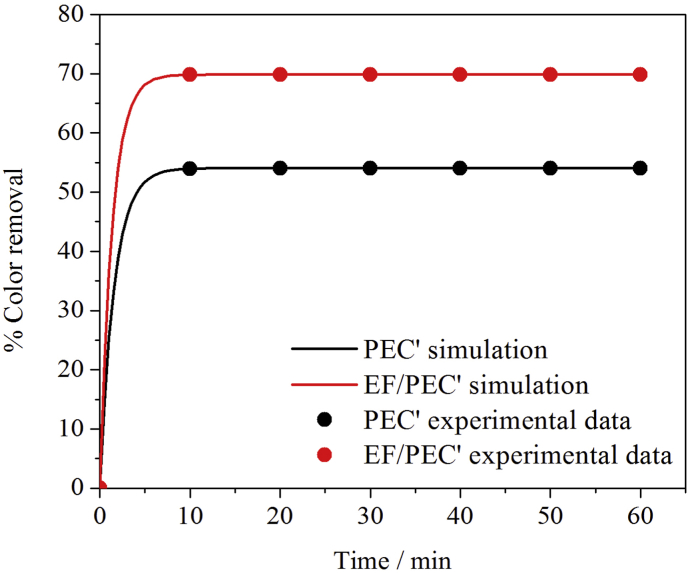
Percentage of color removal (%CR) of MO for PEC′ and EF/PEC′ experiments using the reactor shown in Fig. 1-B. While the circles denote the experimental data points, the solid lines correspond to simulations performed using equation (16). The experiments were carried out using 3 V for electrical polarization, UV light at λ = 365 nm, a MO-saturated AC packed bed cathode and a cuvette modified with a TiO_2_/Ti_4_O_7_ film as photo-anode. (For interpretation of the references to color in this figure legend, the reader is referred to the Web version of this article.)

In this context, Fig. 7 shows the color removal percentage (*%CR*) of a MO solution obtained from experiments carried out in the absence (PEC′) and in the presence (EF/PEC′) of Fe(II) species using a TiO_2_/Ti_4_O_7_/PMMA anode and an AC packed cathode previously saturated with the MO dye (see Fig. 1-B). Both curves reveal a fast increasing *CR* within the first 10 min. Afterwards, the rate of *CR* change is substantially smaller, resembling a quasi-stationary type behavior that reaches *CR* percentages of 53 for PEC′ and 71% for EF/PEC’. These two limiting values show that at long times, the decolorization processes (PEC′, adsorption and EF when Fe(II) ions are present) are compensated with dye desorption events from the previously saturated AC. Comparison of these two values on the other hand, suggests that the EF contribution to the decolorization processes accounts for about 30% of the total color removal observed at 60 min for EF/PEC’.

**Fig. 8:**
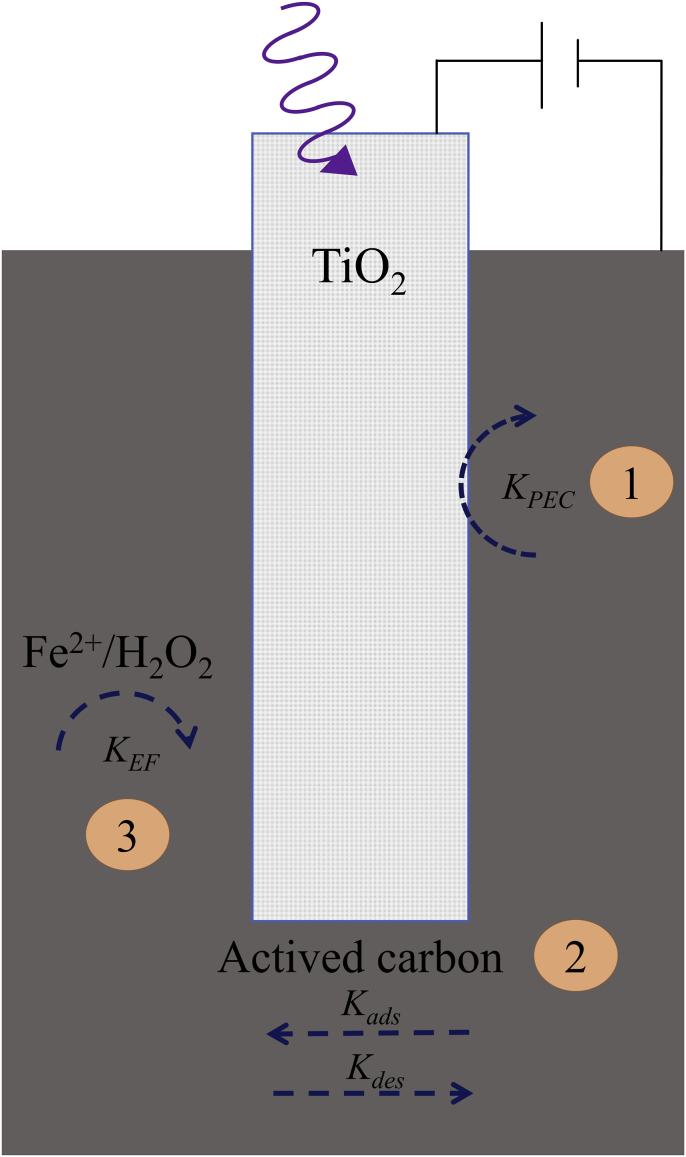
Schematic representation of the MO color changing processes that take place in the photoelectrochemical reactor under study. (1) photoelectrochemical contribution in the anode (PEC′), (2) MO dye adsorption/desorption (AD) processes on the surface and pores of the AC packed bed electrode, and (3) EF in the vicinity of the cathode surface. (For interpretation of the references to color in this figure legend, the reader is referred to the Web version of this article.)

In the reactor shown in Fig. 1-B, there is a semiconductor based anode and an AC packed bed that not only works as a cathode for oxygen reduction but also as an adsorbent material from which dye molecules are simultaneously being taken from and released to the electrolytic solution [54]. Therefore, as can be seen in Fig. 8, there is a complex combination of processes taking place in the photoelectrochemical reactor under study. From the perspective of MO dye absorbance change, i.e., color removal, these processes can be simplified as follows: (1) photoelectrochemical induced decolorization at the anode surface (PEC’, which would account for electrooxidation and photocatalytic contributions), (2) Simultaneous adsorption and desorption processes of dye molecules at the surface and pores of the particles of the AC packed bed (AD) and (3) Fenton induced decolorization of the dye species at the cathode surface (EF).

Under this simplified scheme, equation (8) shows that the rate of mass change (*dM*_*sol*_*/dt*) of the dye in solution (which is what can be experimentally observed trough absorbance measurements) can be expressed in terms of MO mass changes due to adsorption and desorption taking place on the surface of AC (*dM*_*AD*_*/dt*) and to PEC’ (*dM*_*PEC’*_*/dt*) and EF (*dM*_*EF*_*/dt*) dye degradation processes.

(8) $$\frac{dM_{sol}}{dt}=\frac{dM_{AD}}{dt}+\frac{dM_{PEC\text{’}}}{dt}+\frac{dM_{EF}}{dt}$$

Assuming pseudo-first order kinetics for all the processes described in the right hand side of equation (8) and considering that (*dM*_*AD*_*/dt*) describes two processes (related to adsorption and desorption of MO), it is possible to write equations (9), (10), (11)),

(9) $$\frac{dM_{AD}}{dt}=K_{des}M_{sur}-K_{ads}M_{sol}$$

(10) $$\frac{dM_{PEC’}}{dt}=-K_{PEC’}M_{sol}$$

(11) $$\frac{dM_{EF}}{dt}=-K_{EF}M_{sur}$$

This set of relationships shows that *dM*_*sol*_*/dt* depends on four relevant kinetic constants (*K*_*PEC’*_, *K*_*EF*_, *K*_*ads*_ and *K*_*des*_) and on the mass of the dye in solution (*M*_*sol*_) and on the surface of the AC substrate (*M*_*sur*_). The signs of the kinetic constants on the other hand, show that while there are three negative constants corresponding to dye consuming processes (-*K*_*PEC’*_, -*K*_*EF*_, -*K*_*ads*_), there is also a positive kinetic constant (*K*_*des*_) that describes dye desorption from the originally saturated AC packed bed*.*

The fraction of dye in solution on the other hand, corresponds to *M*_*sol*_/*M*_*sol,0*_ and since the mass of dye on the adsorbent surface under saturation conditions at the beginning of the experiment is rather high, the concentration of *M*_*sur*_ can be assumed to be approximately equal to *M*_*sur,0*_. Under these assumptions equation (8) can be written as,

(12) d(MsolMsol,0)t=(−Kads−KPEC’)MsolMsol,0+(Kdes−KEF)Msur,0Msol,0

where the constants *k*_*1*_, *k*_*2*_ and *k*_*3*_ can be defined as described by (13), (14), (15)),

(13) $$k_{1}=-K_{ads}-K_{PEC’}$$

(14) $$k_{2}=K_{des}-K_{EF}$$

(15) $$k_{3}=\frac{M_{sur,0}}{M_{sol,0}}$$

The solution of equation (12) results in 16 (which strictly applies for EF/PEC′ but can be simplified for PEC’ by removing the EF contribution, i.e., when *k*_*2*_*=K*_*des*_) in which the fraction of mass of MO dye (which can be approximated to the decolorization fraction of the solution) is observed to depend on time in an exponential way.

(16) $$\frac{M_{sol}}{M_{sol,0}}=\left(1+\frac{k_{2}k_{3}}{k_{1}}\right)e^{k_{1}t}-\frac{k_{2}k_{3}}{k_{1}}$$

A first inspection of equation (16) shows apparent consistency with the processes described in Fig. 8. In this way, while *M*_*sol*_ = *M*_*sol,0*_ when *t* = 0, (*M*_*sol*_*/M*_*sol,0*_) at *t*→∞ becomes -(*k*_*2*_*k*_*3*_/*k*_*1*_) which should be a value between 0 and 1. In this context, (*k*_*2*_*k*_*3*_/*k*_*1*_) can be anticipated to be negative since *k*_*1*_ is negative (the sign of *K*_*PEC’*_ and *K*_*ads*_ are both negative) and *k*_*2*_ and *k*_*3*_ should be positive since *k*_*3*_=(*M*_*sur,0*_/*M*_*sol,0*_)>0 and *K*_*des*_ should be larger than *K*_*EF*_ (otherwise a quasi-stationary experimental response at long times would not be observed).

In order to test these ideas and to find out how well the processes schematized in Fig. 8 describe the experimental data, the fraction of color removal values of MO (*CR*, which corresponds to 1- (*M*_*sol*_/*M*_*sol,0*_)) in Fig. 7 were fitted to the simplified kinetic model described by equation (16). The resulting parameters *k*_*1*_ and *k*_*2*_ were computed in this way (*k*_*3*_ was already known since it corresponds to the ratio of *M*_*sur,0*_ and *M*_*sol,0*_ values) and used along with the previously determined experimental value of *K*_*PEC’*_ to calculate the remaining kinetic constants *K*_*ads*_, *K*_*des*_ and *K*_*EF*_*.* The complete set of values thus obtained is shown in Table 1 where it can be seen that for the EF/PEC′ process, *k*_*2*_ is in fact positive (*K*_*des*_-*K*_*EF*_ = 0.023 min^−1^) and since *k*_*1*_ is negative (*k*_*1*_ = −1.870 min^−1^), the ratio (*M*_*sol∞*_*/M*_*sol,0*_) = -(*k*_*2*_*k*_*3*_/*k*_*1*_) = 0.296; a value that as expected, not only agrees well with the experimentally observed *%CR* in Fig. 7, but that also predicts the quasi-stationary behavior of the system at long times (*CR* = 0.70). For the PEC′ process on the other hand, the experiment was carried out in the absence of Fe(II) species and therefore *K*_*EF*_ = 0. Under these conditions, consistency of the fitted parameters is retained since *k*_*2*_ is still positive (in fact larger than for the EF/PEC′ case) and *k*_*1*_ negative. The decolorization limit for PEC’ at long times (*M*_*sol∞*_*/M*_*sol,0*_) = -(*k*_*2*_*k*_*3*_/*k*_*1*_), corresponds in this case to 0.45 and the *CR* to 0.549; a value that is in agreement with a process carried out in the absence of Fe(II) ions; thus reflecting the EF contribution to the process.

**Table 1:** Experimental values for the mass of dye in the solution and at the AC surface at the beginning of the experiment and kinetic constants, *K*_*i*_, of equation (16) that fit the experimental data in Fig. 7.

Kinetic Constant, *K*_*i*_	*K*_*i*_/min^−1^	*R*^*2*^
*K*_*ads*_	1.858	0.999
*K*_*des*_	3.5 × 10^−2^	0.999
*K*_*EF*_	1.23 × 10^−2^	0.998
*K*_*PEC’*_	1.21 × 10^−2^	–

*M*_*sur,0*_ (mg) = 16.41

*M*_*sol,0*_ (mg) = 0.681

Simulated curves for both experiments using equation (16) and the constants in reported in Table 1, are shown in Fig. 7 as continuous lines. Inspection of the shape of these curves in the short time region, reveals that both simulations predict a rapid increase in the %*CR* that becomes a quasi-stationary response at rather short times (t < 2 min). In the 2<*t* < 60 min time window on the other hand, the data in Fig. 7 not only suggests different limiting *CR* values (0.549 and 0.7 for PEC′ and EF/PEC′, respectively) defined by a process in which the rate of color change for the two experiments under study is balanced by dye-consuming (adsorption, PEC′ and, when Fe(II) is present, EF) and dye-“generating” (desorption from the previously saturated AC material) processes that are simultaneously taking place in the reactor. By grouping the constants associated to these two types of opposing effects in dye-concentration increasing and decreasing rate constants (*K*_*increase*_ and *K*_*decrease*_), it turns out that while *K*_*increase*_ = 0.035 min^−1^ for both processes (as it should be since dye desorption occurs in EF/PEC′ and PEC′ processes in exactly the same way), *K*_*decrease*_ = 1.858 and 1.882 min^−1^ for PEC′ and EF/PEC’, respectively. The difference between the *K*_*decrease*_ values for the two processes under study is obviously related to *K*_*EF*_ (the contribution of the electro-Fenton decolorization process) and in this context, it is interesting to note that the small value of this difference is readily translated into the larger effect for the limiting %*CR* levels observed in Fig. 7, due to the different mass values of the dye that must be used to compute the corresponding decolorization rates (in this case, *M*_*sur*_ and *M*_*sol*_ are different in at least one order of magnitude).

## 4 Conclusions

In this work, films of TiO_2_, Ti_4_O_7_ and a 1:1 TiO_2_/Ti_4_O_7_ mixture, were prepared on the external surface of PMMA spectroscopy cuvettes and later, characterized in terms of their structure, photocatalytic and electrochemical activity towards the decolorization of a MO dye aqueous solution. The results of photo-, electro- and photoelectrochemical experiments, revealed that while TiO_2_ and Ti_4_O_7_ perform better in terms of photocatalytic and electrocatalytic activity, respectively, the TiO_2_/Ti_4_O_7_ mixture combines the properties of the two materials, thus becoming the best semiconductor film for the photoelectrochemical MO degradation tests surveyed.

The preparation of the TiO_2_/Ti_4_O_7_ composite photoanode on the external surface of PMMA cuvettes allowed the illumination of the semiconductor film from within the cuvette, making the immersion of this electrode in an AC packed bed possible. This approach, which will be extended for the use of semiconductor modified optic fibers [31] immersed in carbon particle based cathodes, was roughly tested in this work with a 3D-type electrochemical reactor in which electro-Fenton events on the surface of the polarized AC packed bed were successfully coupled to the photoelectrochemical anodic degradation process.

The complex mixture of events that take place within the reactor gave rise to efficient color removal performances that reached values close to a 70% in 60 min. Using a simplified model, the performance of the reactor could be successfully fitted to the associated rate constants of the corresponding processes, opening up the possibility for the future design of a novel photo-electro-Fenton 3D reactor in which the adsorption, photo-anodic and electro-Fenton cathodic processes, as well as the area/volume ratio, could be optimized.

## Declaration of competing interests

The authors declare that they have no known competing financial interests or personal relationships that could have appeared to influence the work reported in this paper.
